# Assessment of the Reversion to Virulence and Protective Efficacy in Pigs Receiving the Live Attenuated Classical Swine Fever Recombinant Vaccine Candidate FlagT4G

**DOI:** 10.3390/vaccines13050544

**Published:** 2025-05-20

**Authors:** Elizabeth Ramirez-Medina, Lauro Velazquez-Salinas, Alyssa Valladares, Ayushi Rai, Leeanna Burton, Leandro Sastre, Ediane Silva, Guillermo R. Risatti, Llilianne Ganges, Manuel V. Borca

**Affiliations:** 1 Plum Island Animal Disease Center, Agricultural Research Service, United States Department of Agriculture, Orient Point, NY 11944, USA; lauro.velazquez@usda.gov (L.V.-S.); alyssa.valladares@usda.gov (A.V.); ayushi.rai@usda.gov (A.R.); leeanna.burton@usda.gov (L.B.); leandro.sastre@usda.gov (L.S.); ediane.silva@usda.gov (E.S.); 2 Oak Ridge Institute for Science and Education (ORISE), Oak Ridge, TN 37830, USA; 3 Department of Pathobiology, University of Connecticut, 61 North Eagleville Road, Storrs, CT 06269, USA; guillermo.risatti@uconn.edu; 4 IRTA, Programa de Sanitat Animal, Centre de Recerca en Sanitat Animal (CReSA), Bellaterra, 08193 Barcelona, Spain; llilianne.ganges@irta.cat; 5 WOAH Reference Laboratory for Classical Swine Fever, IRTA-CReSA, 08193 Barcelona, Spain

**Keywords:** classical swine fever virus, live attenuated vaccines, DIVA vaccine, FlagT4G

## Abstract

**Background/Objectives**: Control of classical swine fever virus (CSFV) in endemic countries relies on vaccination using live attenuated vaccines (LAVs). Most of these LAVs do not allow for the differentiation of vaccinated animals from infected animals (DIVA) based on their serological response. FlagT4G vaccine is a novel candidate that confers robust protective immunity early after vaccination and shows DIVA capabilities. **Methods**: This report presents the characterization of FlagT4G virus in terms of the stability of its genomic and attenuated phenotypes assessed by a reversion to virulence protocol, as well as its protective efficacy by determining the minimal protective dose. **Results**: Results presented here demonstrate that after five consecutive passages in groups of 5-week-old susceptible domestic pigs, FlagT4G virus remains genetically stable, and its attenuated phenotype remains unaltered. In terms of efficacy, FlagT4G virus induced solid protection against the intranasal challenge with 10^5^ tissue culture infectious dose (TCID_50_) of virulent field isolate Brescia virus, even with a vaccine dose as low as 10^2^ TCID50. **Conclusions**: Results presented here indicate that the FlagT4G vaccine may be a useful tool for CSFV control.

## 1. Introduction

Classical swine fever is a highly contagious and economically devastating viral swine disease [1] caused by classical swine fever virus (CSFV), a pathogen belonging to the Flaviviridae family and genus Pestivirus [2]. CSFV is associated with a single serotype from which three genotypes and diverse subtypes have been described [2]. The devastating economic consequences of CSF can be exemplified in the Netherlands during the 1997/1998 outbreak, where the economic losses were estimated to be about $2.3 billion. According to the World Organization for Animal Health (June, 2024, Classical swine fever—WOAH—World Organization for Animal Health), 37 countries are recognized as “CSF free members”, three countries (Brazil, Colombia and Ecuador) are recognized as “members having a zone free of CSF”, and one country, Kazakhstan, has had its free CSF status suspended. Conversely, in several countries in Asia, Africa, and Europe, the actual status of the disease is unknown, a situation that is consistent with previous reports back in 2017 [2], indicating the real threat that CSF represents for the pork industry worldwide.

Currently, vaccination appears to be one of the best tools to control CSF in affected countries. Overall, live attenuated vaccines (LAVs) have shown a superior performance over subunit-based vaccines [3]. At this point, several potential CSF LAVs have been developed and are in constant evaluation [4,5,6], including the widely used C-Strain [3], representing different options to control CSFV.

As a result of our research program oriented to determine critical sites in the genome of CSFV implicated in the virulence in pigs [7,8], we have developed the CSF LAVs candidate FlagT4G virus [9,10]. FlagT4G virus is a recombinant derivative of the highly virulent CSFV strain Brescia [9] produced by the introduction of a 19-residue insertion at the carboxyl terminus of the E1 glycoprotein (harboring the Flag epitope) [7], as well as a specific number of residues mutations at a highly conserved CSFV epitope in the E2 glycoprotein recognized by monoclonal antibody (mAb) WH303 [8,11]. In this context, FlagT4G virus possesses a positive antigenic marker (synthetic Flag^®^ epitope) as well as a negative marker (absence of the WH303 epitope) [9]. Each of these antigenic markers constitutes the molecular basis for developing potential serological tests to differentiate infected from FlagT4Gv vaccinated animals (DIVA). In fact, a DIVA ELISA test has been developed based on the lack of reactivity with the WH303 epitope in animals vaccinated with the FlagT4G virus [12].

Importantly, vaccination with the FlagT4G virus induces effective protection against the challenge with high virulent CSFV as early as 3 days post-vaccination [9]. Although not completely proven, it appears that a strong induction of interferon could be responsible for this early protection at a time when the CSFV-specific adaptive immune response, including the antibody response, is not yet detected [13,14].

In this study, we characterized the stability of the genome and attenuated phenotype of the FlagT4G virus using a protocol to assess the reversion to virulence, as well as its protective efficacy by assessing its minimal protective dose in domestic pigs.

## 2. Materials and Methods

### 2.1. Virus and Cell Cultures

Recombinant vaccine candidate FlagT4G virus was developed by genetic manipulation of the CSFV Brescia isolate using reverse genetics as previously described [9]. Brescia Infectious Clone virus (BICv) is a virus derived from an infectious clone encoding for the highly virulent strain Brescia using reverse genetics [8]. Primary cultures of swine macrophages and of the cell line SK6 were produced as previously described [7]. Virus titers were calculated in SK6 cells and expressed in tissue culture infectious dose (TCID_50_) exactly as previously described [7]. Differential staining of the FlagT4G and BICv was performed as previously described [10] by immunocytochemistry using monoclonal antibodies specifically recognizing the presence and absence of the Flag and the WH303 epitopes, respectively.

### 2.2. Virus Sequencing

The whole genome sequence of the FlagT4G virus was obtained using an enhanced multiplex tiling RT-PCR protocol to amplify the complete coding sequence of the virus. A multiplexed paired-end sequencing library of the PCR products was generated using the Nextera DNA Flex Library Prep Kit (Illumina, Foster City, CA, USA). The library was adjusted to 100 pM, and 5% of the PhiX control (Illumina) was added to the library. The library pool was loaded into an ISeq100 i1 Reagent Kit (Illumina). The barcoded multiplexed library sequencing (2 × 151 bp) was performed on an Illumina iSeq100 platform. Fastq files were uploaded to Galaxy [15] and utilized in batch mode to recognize and run pairs together. Residual adapters and bases with low-quality scores were removed using Trimmomatic version 0.38.0 [16], removing bases from each read with a quality score < Q 20 and requiring a minimum read length of 50 bases each.

### 2.3. Reversion to Virulence Protocol

Five groups of five animals each (5–6 week old European cross-bred animals) were used to evaluate the stability of the attenuated phenotype of the FlagT4G virus. The first group of animals was intramuscularly (IM) inoculated with 10^5^ TCID_50_ of FlagT4G virus. Presence of clinical signs, including body temperature, was observed daily for 7 days post inoculation (dpi). At 7 dpi, all animals were euthanized, their tonsils removed, macerated, and resuspended at 10% weight/volume in DMEM (Gibco, Thermo-Fisher, Waltham, MA, USA) containing 10% of irradiated fetal calf serum (HyClone, South Logan, UT, USA). The obtained suspension (1 mL per animal) was immediately used to IM inoculate a second group of animals. This second group was treated similarly to the first one in terms of observation and sample processing. The procedure was repeated with the third and fourth groups. The fifth group was treated in the same way, with the exception that the period of clinical observation was extended to 21 dpi.

### 2.4. Minimal Protective Dose Protocol

Five groups of five animals each (European cross-breed, between 15 and 20 kg) were used to evaluate the protective efficacy of FlagT4G. Each of the four groups was IM inoculated with a different dose of FlagT4G virus, ranging from 10^2^ to 10^5^ TCID_50_, respectively. The fifth group remained as a control. Presence of clinical signs, including body temperature, was observed daily for 28 days post-vaccination (dpv). All groups were challenged intranasally (IN) at 28 days post-vaccination with 10^5^ TCID_50_ of BICv, a virus derived from an infectious clone encoding for the highly virulent strain Brescia [8]. Presence of clinical signs, including body temperature (along with depression, anorexia, cough, diarrhea, skin coloration, vomiting, nervous signs) was observed daily for 21 days post challenge (dpc).

## 3. Results

### 3.1. Reversion to Virulence Experiment

#### 3.1.1. Clinical Signs

The stability of the attenuated phenotype of the FlagT4G virus was evaluated by performing a reversion to virulence experiment. Basically, the FlagT4G stock virus was successively passaged five times between groups of five pigs aged between 5 and 6 weeks, and the level of attenuation was evaluated daily in all animals in each of the inoculated groups.

**Figure 1:**
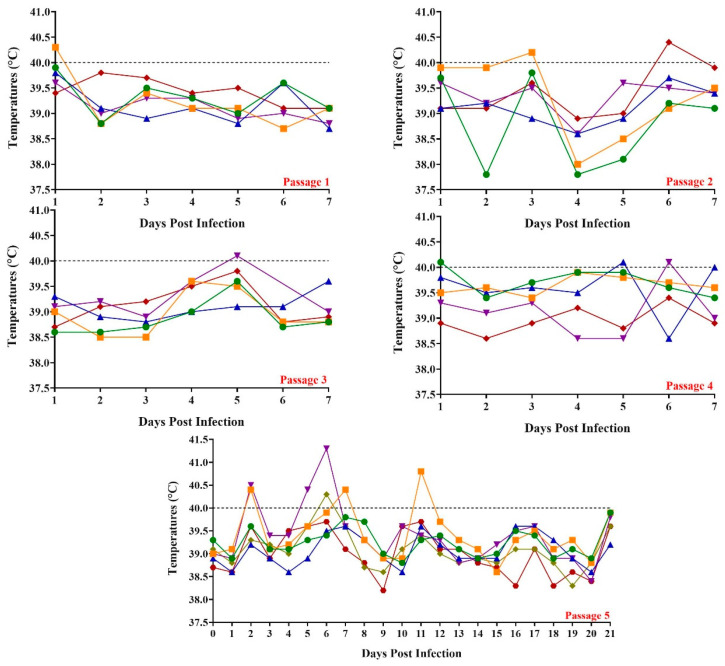
Evolution of body temperature in animals inoculated with FlagT4G during five consecutive passages (panels Passage 1–5, respectively). Data are presented as individual values (expressed as °C) for each of the inoculated animals in each of the different groups. The color lines represent the data of each animal.

The first group of animals was inoculated IM with 10^5^ TCID_50_ of FlagT4G virus (Figure 1). Animals did not show any clinical signs associated with CSF, and all animals remained with rectal temperatures below 40 °C (which we considered the top physiological value of body temperature), with the exception of a single animal showing a slight increase only on 1 dpi.

The second group of animals was inoculated with IM with a suspension of the virus obtained from a pool of tonsils from animals in the first group, which was obtained at the seventh dpi. The titer of the suspension was 10^2^ TCID_50_/mL. All animals in this second passage remained clinically normal during the seven-day observational period, with rectal temperatures below 40 °C, with the exception of two animals that presented only one day with temperature values slightly over that limit at 2 and 5 dpi, respectively (Figure 1). It should be noted that no other additional clinical sign was associated with these two minor transitory temperature increases. The third and fourth groups of animals had a similar behavior to the second group. Inoculum titers, also IM inoculated, were 10^3.2^ and 10^2.1^ TCID_50_/mL for the third and fourth groups, respectively. All inoculated animals in these two groups remained clinically normal during the seven-day observational period. In both groups, rectal temperature values remained below 40 °C except for one animal in the third group and three animals in the fourth group, showing temperature values just over the 40 °C limit by only one day (Figure 1). Again, no additional clinical sign was associated with this slight and transitory temperature increase.

The animals in the last group, which received each of them IM 10^3.2^ TCID_50_/mL of virus suspension obtained from animals in group four, also remained clinically normal during the 21 dpi. In addition, temperature remained below 40 °C with the exception of a day increase in three of the five animals in the group. One of them showed mild increases on days 2, 8, and 11 pi, with values below 41 °C, a second animal on day 6 pi (below 40.5 °C), and a third animal on days 6–7 with values below 41.5 °C. Again, no additional clinical sign was associated with these slight and transitory temperature increases during the 21-day observational period (Figure 1).

Therefore, evaluation of the FlagT4G virus using the described reversion to virulence methodology demonstrated that the vaccine candidate remains stable in its attenuated phenotype.

#### 3.1.2. Antigenic Profile of FlagT4G

**Figure 2:**
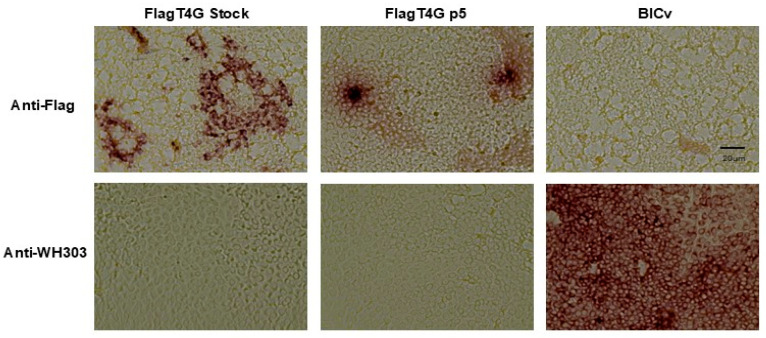
Antigenic profile of FlagT4G virus after the consecutive passages in the reversion to virulence experiment (FlagT4Gv p5). Reactivity of the FlagT4Gv p5 with monoclonal antibody specific for Flag and (Anti-Flag mAb) and CSFV E2 epitope recognized by monoclonal antibody WH303 (WH303 mAb) compared with the stock FlagT4G virus (FlagT4Gv) and BICv.

The FlagT4G virus harbors two antigenic markers that differentiate the vaccine candidate from the virus field strains. One is the Flag positive antigenic marker and the other is the negative antigenic marker based on the absence of the epitope recognized by the mAb WH303 [10]. The stability of the antigenic profile of the FlagT4G virus after the five sequential passages of the virus through the different groups of animals in the reversion to virulence protocol was analyzed based on the recognition of these epitopes with the anti-Flag and WH303 reagents. Immunocytochemistry studies demonstrated that the FlagT4G virus obtained from animals in the fifth group preserves its antigenic profile, strongly reacting with the Flag epitope and lacking any reactivity with the mAb WH303, similar to the FlagT4G stock virus’ performance (Figure 2).

#### 3.1.3. Genetic Stability of FlagT4G

To evaluate the genetic stability of the FlagT4G during its successive passages among the different groups of pigs in the reversion to virulence study, the full-length sequence of the virus genome was analyzed in the virus population obtained in each of the passages. Comparison of the genome sequence obtained after the first passage in animals with that of the original stock virus does not show any detectable change. After the second passage, there was an amino acid substitution (Phe to Iso) at position 851 of the polyprotein, within the structural protein E2. During the third passage, an additional residue change occurred (Cys to Tyr) at position 438 of the CSFV polyprotein, within the structural protein E0. During the fourth passage in pigs, there was one additional amino acid substitution at position 3007 of the polyprotein, within the nonstructural protein NS5A genome, where a Lys was replaced by an Arg. During the fifth passage in animals, there were no amino acid changes. No nucleotide changes occurred in the untranslated regions of the virus genome during the successive passages of FlagT4G in pigs.

Therefore, during the five passages in animals, the FlagT4G virus incorporated three amino acid residue substitutions. The residues’ substitutions are quite conserved in terms of their characteristics, as Phe to Iso substitution involved residues with hydrophobic characteristics, and Lys to Arg replacement involved two polar positive charge residues. As already shown, none of these residue substitutions affected the antigenic profile, particularly in terms of the epitopes involved in the DIVA markers Flag and those recognized by the mAb WH303.

#### 3.1.4. Determination of Minimal Protective Dose

To determine the protective efficacy of the FlagT4G virus, the minimal dose of the virus that is able to protect animals against the challenge of the parental highly virulent BIC virus was calculated. Five groups of six pigs each (with a body weight ranging from 30 to 40 lbs) were IM inoculated with different doses (from 10^2^ to 10^5^ TCID_50_) of FlagT4G virus, or mock inoculated. Animals were clinically evaluated daily for 28 days after inoculation, including changes in their rectal temperature, in order to detect potential local or general toxicity of the vaccine.

**Figure 3:**
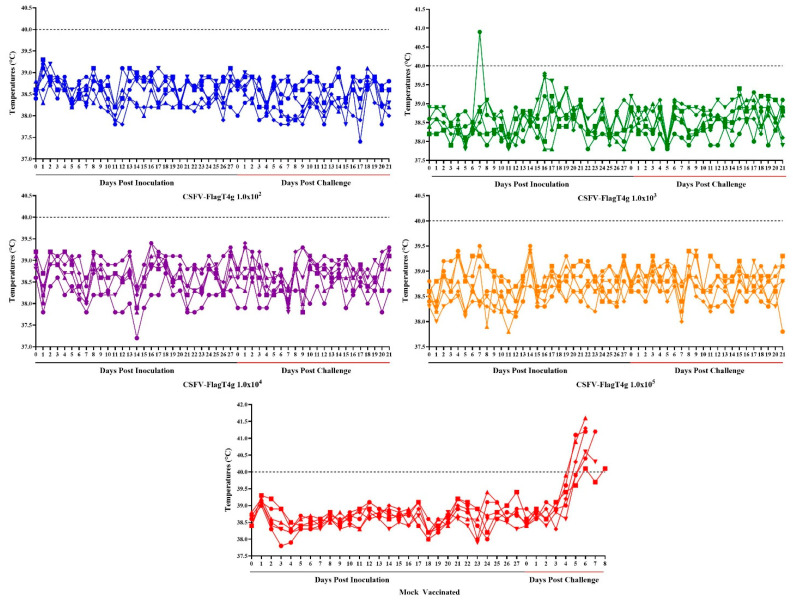
Kinetics of body temperature values in pigs intramuscularly inoculated with different doses 10^2^–10^5^ TCID_50_) of FlagT4G virus or Mock Vaccinated before (Days post-inoculation) and after intranasal challenge (Days post-challenge) with 10^5^ TCID_50_ of the virulent parental virus BICv. Each curve represents individual data from each of the animals under each of the treatments. The color lines represent the data of each group.

All animals in all four inoculated groups remained clinically normal without any clinical signs that may be associated with CSF. Rectal temperatures of all inoculated animals remained well below 40 °C with only one exception, an animal in the group receiving 10^4^ TCID_50_, which presented only one day (at 7 dpi) with 41 °C without the presence of any additional clinical sign (Figure 3). In fact, clinical presentation and rectal temperature in all FlagT4G inoculated animals did not substantially differ from those parameters recorded in the mock inoculated group. These data demonstrate that inoculation of the FlagT4G virus does not cause any clinical problems in the vaccinated animals.

The protective efficacy of the different doses of FlagT4G virus was evaluated after challenge of all FlagT4G vaccinated animals using 10^5^ TCID_50_ of the highly virulent parental BIC virus by IN route. The appearance of CSF-related clinical signs (including an increase in rectal temperature) was monitored daily for 21 dpi.

**Figure 4:**
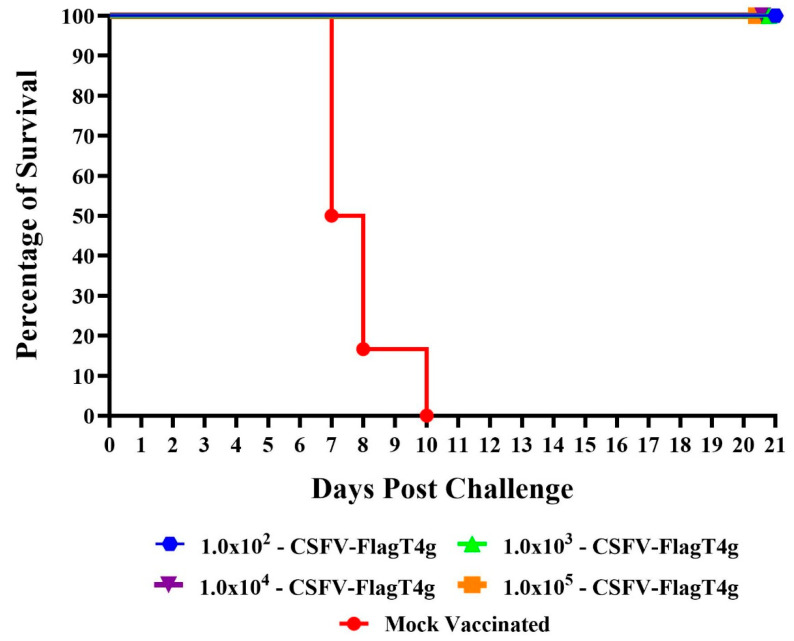
Evolution of mortality in pigs intramuscularly inoculated with different doses 10^2^–10^5^ TCID_50_) of FlagT4G virus or Mock Vaccinated and challenged with 10^5^ TCID_50_ of the virulent parental virus BICv.

All animals in all the vaccinated groups remained clinically normal without showing any clinical sign that can be ascribable to CSF. None of the animals in any of the vaccinated groups developed a rectal temperature reading at or over 39.5 °C during the 21-day observational period. Conversely, three of the control animals presented rectal temperatures over 40 °C by day 5 pi, with all animals having temperature values over 40 °C by day 6 pi, developing severe forms of the disease, leading to euthanasia of all of them between day 6 and 8 pi (Figure 3 and Figure 4).

The presence of the BIC virus in blood was assessed as a parameter for evaluating protection against the infection in the challenged animals at 7 dpc. Presence of BIC virus was specifically detected by its reactivity to the mAb WH303. BIC virus was detected at titers ranging between 10^4.05^ and 10^6.55^ TCID_50_/mL in all control animals but was systematically absent in all vaccinated animals inoculated with different doses of FlagT4G virus. These results suggest that protection against the challenge in the FlagT4G vaccinated animals not only prevented the presentation of clinical disease but also may prevent the replication of the challenge virus, even at the lowest dose (10^2^ TCID_50_) of FlagT4G tested.

## 4. Discussion

Results reported here demonstrate that the FlagT4G vaccine candidate presents a solid attenuated phenotype along with a stable genome. The genome of the FlagT4G virus suffers a small number of nucleotide point mutations, leading to a very limited number of amino acid substitutions along the whole genome. Some sporadic and mild increases in body temperature were detected in some of the animals, particularly in those of the fifth group. In this regard, it should be stressed that (i) in no case did the temperature increases last more than a day, (ii) in most of the cases temperature values were not higher than 40.5 °C and only in one case was it barely higher than 41 °C, and (iii) in no case, were these sporadic temperature increases accompanied by any additional clinical signs. Therefore, no signs of clinical disease (except for that brief, isolated, and very mild rise in body temperature) were seen in any animal in any of the five groups. In addition, it is important to consider that animals involved in passages 1 to 4 were only observed for 7 days while those in passage 5 were observed for 21 days, increasing the opportunity of those sporadic, and transient mild increases in body temperature to occur.

It is difficult to evaluate the potential involvement of the amino acid residue substitutions detected in the virus obtained from passage five in a hypothetical increase in virulence. Deletion of Cysteine at position 438 of the CSFV polyprotein, within the structural protein E0, has been involved in the formation of protein dimers and has been related to attenuation of CSFV virulence in pigs [17]. Therefore, mutation of this residue should produce attenuation rather than an increase in virulence. The Phenylalanine residue at position 851 of the polyprotein is located in an area of the structural protein E2, which has been involved in virulence along with five other adjacent amino acid residues [8]. Residue substitutions in all six positions have been shown to be involved in a change in virulence. To our knowledge, the Lysine residue at position 3007 of the polyprotein, within the nonstructural protein NS5A genome, has not been involved in CSFV virulence in pigs. Importantly, none of these changes altered the reactivity with the antibodies recognizing the modified epitopes (Flag and WH303), which are the bases of the differential antigenic profile that provides FlagT4G virus its DIVA capability. Comparable studies were performed with other live attenuated CSFV vaccine strains employing similar methodological protocols [18]. For instance, reversion to virulence studies using the live attenuated vaccine GPE^−^, developed and used in Japan, showed that the virus remains in the tonsils during the passages with a slight increase in virus titers along the five passages. In the same study, more attenuated recombinant versions of the GPE^−^ strain became undetectable in tonsils in the earlier passages of the protocol [18]. Further testing of the FlagT4G vaccine may need to be performed to assess its safety profile following safety requirements (safety in young animals and in pregnant sows, the evaluation for transplacental transmission, assessment of non-transmissibility, etc.) described by the WOAH Terrestrial Manual (https://www.woah.org/fileadmin/Home/fr/Health_standards/tahm/3.09.03_CSF.pdf, 25 May 2025).

Regarding its efficacy, FlagT4G virus was shown to induce solid protection against the appearance of clinical disease in animals after challenge with a high dose of highly virulent CSFV strains such as Brescia or Margarita [9,13,14]. Interestingly, besides preventing the development of clinical disease, based on the absence of challenge virus in blood samples of the vaccinated/challenge animals, it appears that FlagT4G aborts or greatly reduces the replication of the challenge virus, even in those animals receiving the lowest vaccine dose.

In addition to the positive features described in this report, FlagT4G virus possesses other beneficial characteristics such as inducing solid protection as early as the third day post vaccination [9,13,14]. This protective effect is probably mediated by stimulating early innate immune mechanisms [13,14]. Also, FlagT4G virus possesses a unique characteristic of harboring two strong antigenic markers, which are the bases of the potential development of serological-based DIVA assays [9,10]. Proof of concept ELISA tests have already reported detecting differential serological responses between FlagT4G vaccinated and CSFV infected animals for both the positive and the negative antigenic markers [9,10,14], the Flag and the WH303 epitope, respectively. Negative antigenic markers are critical in the development of DIVA tests, differentiating vaccinated and infected domestic animals, while a positive antigenic marker is essential in the serological identification of vaccinated wild swine.

Therefore, the FlagT4G virus has the potential to be considered a safe and efficacious live attenuated DIVA-compatible CSF vaccine candidate both in endemic situations and in the emergency management of a disease outbreak in CSF-free areas.

## Data Availability

All data are included in the manuscript.
